# Comparative Cost-Effectiveness of Stereotactic Body Radiation Therapy Versus Intensity-Modulated and Proton Radiation Therapy for Localized Prostate Cancer

**DOI:** 10.3389/fonc.2012.00081

**Published:** 2012-08-20

**Authors:** Anju Parthan, Narin Pruttivarasin, Diane Davies, Douglas C. A. Taylor, Vivek Pawar, Akash Bijlani, Kristen Hassmiller Lich, Ronald C. Chen

**Affiliations:** ^1^Health Economics and Outcomes Research OptumInsight; ^2^Accuray Incorporated; ^3^Bayer (Formerly with OptumInsight); ^4^University of North Carolina at Chapel Hill

**Keywords:** prostate, cancer, radiation, cost-effectiveness, localized

## Abstract

**Objective:** To determine the cost-effectiveness of several external beam radiation treatment modalities for the treatment of patients with localized prostate cancer. **Methods:** A lifetime Markov model incorporated the probabilities of experiencing treatment-related long-term toxicity or death. Toxicity probabilities were derived from published sources using meta-analytical techniques. Utilities and costs in the model were obtained from publicly available secondary sources. The model calculated quality-adjusted life expectancy and expected lifetime cost per patient, and derived ratios of incremental cost per quality-adjusted life year (QALY) gained between treatments. Analyses were conducted from both payer and societal perspectives. One-way and probabilistic sensitivity analyses were performed. **Results:** Compared to intensity-modulated radiation therapy (IMRT) and proton beam therapy (PT), stereotactic body radiation therapy (SBRT) was less costly and resulted in more QALYs. Sensitivity analyses showed that the conclusions in the base-case scenario were robust with respect to variations in toxicity and cost parameters consistent with available evidence. At a threshold of $50,000/QALY, SBRT was cost-effective in 75% and 94% of probabilistic simulations compared to IMRT and PT, respectively, from a payer perspective. From a societal perspective, SBRT was cost-effective in 75% and 96% of simulations compared to IMRT and PT, respectively, at a threshold of $50,000/QALY. In threshold analyses, SBRT was less expensive with better outcomes compared to IMRT at toxicity rates 23% greater than the SBRT base-case rates. **Conclusion:** Based on the assumption that each treatment modality results in equivalent long-term efficacy, SBRT is a cost-effective strategy resulting in improved quality-adjusted survival compared to IMRT and PT for the treatment of localized prostate cancer.

## Introduction

The National Cancer Institute estimates that 241,740 new cases of prostate cancer will be diagnosed in 2012, along with 28,170 deaths (National Cancer Institute, 2012). The Prostate Cancer Outcomes Study (PCOS), which was initiated by the National Cancer Institute in 1994, noted that 88% of the newly diagnosed prostate cancer cases will be localized disease. Based on a review of the current published literature, there are several options for patients diagnosed with clinically localized prostate cancer including active surveillance, surgery, and radiation therapy. In 2010, the Agency for Healthcare Research and Quality (AHRQ) concluded that insufficient evidence exists to fully assess the superiority of one type of radiation therapy over another for the treatment of localized prostate cancer (Agency for Healthcare Research and Quality, 2010). The objective of this study is to address this gap by developing a decision analysis model that integrates currently available published evidence on the post-treatment incidence of long-term toxicities to compare the incremental cost-effectiveness of three modern external beam radiation therapies for localized prostate cancer: intensity-modulated radiation therapy (IMRT), proton beam therapy (PT), and stereotactic body radiation therapy (SBRT).

Intensity-modulated radiation therapy, which is a commonly used treatment for patients with localized prostate cancer, involves the external delivery of multiple beams of radiation that conform to the shape of the tumor, and where the intensity of each beam can be modulated in order to spare surrounding healthy tissue. IMRT therapy is typically delivered in 40 fractions (i.e., treatment sessions) and requires 8–9 weeks of treatment. PT is delivered externally to a predefined depth, potentially with little radiation delivered beyond that point, thus sparing surrounding healthy tissue. Like IMRT, PT typically is delivered in 40 fractions and requires 8–9 weeks of treatment. A study conducted by Konski et al. (2007) found that PT was not a cost-effective treatment option for localized prostate cancer when compared to IMRT.

Stereotactic body radiation therapy is the use of accurate and image-guided radiation therapy to treat tumors using up to five intense radiation treatments (Martin and Gaya, 2010). Because a higher dose is given in each fraction, image guidance during treatment and an ability to adjust for tumor/target motion is important in order to minimize treatment-related toxicity. Recent studies have indicated that SBRT toxicity levels are comparable to those of other radiation treatment options (Sanda et al., 2008; Chen et al., 2009; Townsend et al., 2011), with long-term (5-year) progression-free survival at 93% (Freeman and King, 2011).

In the current paper we present a Markov model developed to estimate the comparative long-term incremental cost-effectiveness of IMRT, PT, and SBRT. The analyses are conducted using a lifetime horizon from both the perspective of a health care payer as well as a societal perspective that includes the value of patients’ time spent in treatment.

## Materials and Methods

### Overview

Decision-analytic techniques involved analysis of a lifetime cohort Markov model, programmed in Microsoft^®^ Excel, to assess the cost-effectiveness of three radiation treatment modalities for localized prostate cancer: SBRT, IMRT, and PT. Brachytherapy was not included as a comparator in the model because the method of delivery (i.e., requiring anesthesia and invasive) was considered to be significantly different from SBRT, IMRT, and PT. Furthermore, because of the anesthesia requirement, some studies have shown that brachytherapy is more likely to be used with younger men when compared with external beam radiation (Sanda et al., 2008; Chen et al., 2009).

**Figure 1:**
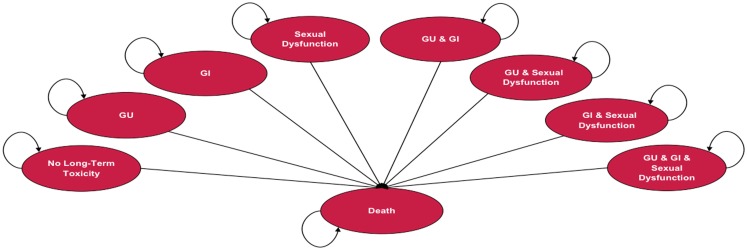
**Markov model**. GI, gastrointestinal; GU, genitourinary; SD, sexual dysfunction.

Depending on the treatment selected, patients in the model are at risk of experiencing one or more of three types of treatment-related long-term toxicity, or death. Long-term toxicities included in the analysis were gastrointestinal (GI), genitourinary (GU), and sexual dysfunction (SD). Long-term toxicity is defined as adverse events = grade 2, using the Radiation Therapy Oncology Group (RTOG) scale ≥12 months following treatment. The model assumes that patients can experience treatment-related long-term toxicity within the first year following treatment and are at risk to continue to experience it throughout their lives. Figure 1 illustrates the Markov states and transitions that can occur in the model. The model includes eight health states that reflect all possible combinations of the three long-term toxicity states (no toxicity, GI only, GU only, SD only, GI & GU, GI & SD, GU & SD, GI & GU & SD). Since data were not available regarding the duration of side effects, the assumption in the model is that during each Markov cycle patients remain in the same Markov health state or die. Measures of utility (an overall quality of life measure on a 0 to 1 scale) and cost are assigned to each state. Treatment-specific mortality and long-term annual mortality rates with localized prostate cancer are based on published sources and data from SEER, respectively. The model assumes that the long-term disease control is comparable across the treatments, which is consistent with currently available literature.

In the base-case we assume that all toxicities are associated with a one-time cost. Analysis of quality-adjusted survival with the Markov model involves tracking patients according to whether or not they experience each of the types of long-term toxicity, and weighting their years of survival by the utility associated with their corresponding toxicity state. The model calculates the quality-adjusted life years (QALYs) and costs per patient over the course of a lifetime. Analyses were performed from a healthcare payer perspective. An additional analysis was conducted from a societal perspective that included the forgone value of patients’ time spent in initial radiation treatment.

### Model parameters and data sources

The model population consists of 65-year-old males with localized prostate cancer who declined or were ineligible for surgery and are eligible for external radiation therapies. Model parameters and data sources are summarized in Tables 1– 5, and are described below.

**Table 1:** **Characteristics of the 10 studies chosen for the meta-analysis**.

Treatment	Study	Sample size	Follow-up time (months)	Age (range)	Toxicity studied
SBRT	Friedland et al. (2009)	112	24	Mean 70 (55–87)	SD
	Katz et al. (2010)	206	17 (dose 36.25)	Mean 69 (45–88)	GU, GI, SD
			30 (dose 35)	
	King et al. (2009)	41	33	Median 66 (48–83)	GU, GI
	Wiegner and King (2010)	20	35.5	Median 68 (57–83)	SD
IMRT	Kirichenko et al. (2006)	928	36	NA	GU, GI
	Zelefsky et al. (2002)	772	36	Median 69 (46–86)	GU, GI, SD
	Zelefsky et al. (2006)	561	96	Median 68 (46–86)	GU, GI, SD
PT	Schulte et al. (2000)	870	36	NA	GU, GI
	Slater et al. (1998)	643	36	NA	GU, GI
	Slater et al. (1999)	315	36	NA	GU, GI

*GI, gastrointestinal; GU, genitourinary; IMRT, intensity-modulated radiation therapy; NA, not available; PT, proton beam therapy; SBRT, stereotactic body radiation therapy; SD, sexual dysfunction*.

#### Long-term toxicity and mortality

The probability of experiencing each type of treatment-related long-term toxicity was derived using meta-analytic techniques. A search was conducted in MEDLINE, EMBASE, PsycINFO, and PubMed for studies published between 1998 and 2010 that reported GU, GI, and SD toxicities over a given period of time. Table 1 displays the characteristics of the 10 studies that were chosen for the meta-analysis. Due to a lack of randomized controlled trials, all 10 studies were either clinical trials with no control group or observational studies with a single cohort. The meta-analysis used a random-effects model to minimize heterogeneity across studies due to clinical or methodological differences. Furthermore, the meta-analysis used the normally distributed log odds ratio as the outcome variable, after which the pooled log odds ratio was converted back to an annual rate.

Table 2 displays the treatment-related probabilities of experiencing the various types of long-term toxicity and mortality. A recent paper published by Coen et al. (2012) suggests that PT toxicity has changed very little in the last 10 years. Also, in the absence of long-term SD toxicity rates for PT, in the base-case, the SD rates were assumed to be equal to IMRT because the method of delivery over time was similar – i.e., small doses over a long period of time. An age-specific mortality rate for prostate cancer patients was applied to all patients in the model using SEER data (Altekruse et al., 2010). The risk of dying from treatment (IMRT, SBRT, and PT) was assumed to be zero.

**Table 2:** **Treatment-related mortality and long-term toxicity**.

Probabilities	Base-case default value	SE	Source
**TREATMENT-RELATED MORTALITY**			
SBRT	0.000	–	Assumption
IMRT	0.000	–	Assumption
PT	0.000	–	Assumption
**LONG-TERM TOXICITIES – SBRT**			
GU	0.040	0.023	Friedland et al. (2009), Katz et al. (2010), King et al. (2009)
GI	0.027	0.010	Katz et al. (2010), King et al. (2009)
SD	0.159	0.088	Friedland et al. (2009), Katz et al. (2010), Wiegner and King (2010)
**LONG-TERM TOXICITIES – IMRT**			
GU	0.035	0.016	Kirichenko et al. (2006), Zelefsky et al. (2002), Zelefsky et al. (2006)
GI	0.013	0.008	Kirichenko et al. (2006), Zelefsky et al. (2002), Zelefsky et al. (2006)
SD	0.272	0.232	Zelefsky et al. (2002), Zelefsky et al. (2006)
**LONG-TERM TOXICITIES – PT**			
GU	0.019	0.004	Schulte et al. (2000), Slater et al. (1998), Slater et al. (1999)
GI	0.015	0.006	Schulte et al. (2000), Slater et al. (1999)
SD	0.272	0.232	Assume the same as IMRT

*GI, gastrointestinal; GU, genitourinary; IMRT, intensity-modulated radiation therapy; PT, proton beam therapy; SBRT stereotactic body radiation therapy; SD, sexual dysfunction; SE, standard error*.

*An RTOG (Radiation Therapy Oncology Group) scale was used to measure toxicity in all but the Kirichenko et al., 2006; scale not reported) and Zelefsky et al., 2006; CTCAE scale) studies*.

#### Health-related quality of life

Quality-adjusted life expectancy associated with each treatment was estimated using utilities assigned to each health state in the model (Table 3). Utilities are weights that quantify health-related quality of life (HRQoL) on a scale from 0 to 1, where 0 reflects death and 1 reflects perfect health. The QALYs associated with each of the long-term toxicity health states were estimated using utility weights derived from Stewart et al. (2005). The utility value for not experiencing treatment-related long-term toxicity was assumed to be 1, and the utility value for death was 0. QALYs in each Markov state and cycle were determined by multiplying the utility associated with each of the long-term toxicities by a baseline age-specific background utility for males in the general population (Hamner et al., 2006).

**Table 3:** **Model utility inputs**.

Utility parameters	Base-case default value	SE	Source
No long-term toxicities	1.00	0.00	Assumption
GU	0.83	0.02	Stewart et al. (2005)
GI	0.71	0.02	Stewart et al. (2005)
SD	0.89	0.01	Stewart et al. (2005)
GU & GI	0.70	0.04	Stewart et al. (2005)
GU & SD	0.79	0.03	Stewart et al. (2005)
GI & SD	0.57	0.04	Stewart et al. (2005)
GU & GI & SD	0.45	0.04	Stewart et al. (2005)
Death	0.00	0.00	By definition

*GI, gastrointestinal; GU, genitourinary; SD, sexual dysfunction; SE, standard error; QALYs in each Markov state and cycle are determined by the product of the baseline age-specific background utility for males (Hamner et al., 2006) and the utility associated with long-term toxicities*.

#### Resource utilization

Table 4 displays the base-case resource utilization parameter values for each treatment, including the numbers of routine and toxicity-related office visits and treatments, and the number of patient hours lost due to treatment. The resource utilization parameter values were used to estimate treatment-related costs in the model.

**Table 4:** **Model resource utilization inputs**.

Utilization parameters	Value	Source
**TREATMENT-RELATED (PER TREATMENT)**		
SBRT		
Work-time lost (hours per treatment)	10	Expert opinion
IMRT		
Work-time lost (hours per treatment)	90	Ollendorf et al. (2009)
PT		
Work-time lost (hours per treatment)	100	Ollendorf et al. (2008)
**ROUTINE MONITORING (PER YEAR)**		
Office visit (1st year)	2	Expert opinion
Office visit (subsequent years)	1	Expert opinion
PSA	2	Expert opinion
**TOXICITY-RELATED**		
GU toxicity (per year)		
Routine office visit	2	Expert opinion
Pharmacologic treatment (oxybutynin)	365	Daily dosage based on package insert
Cystoscopy	1	Expert opinion
GI toxicity (one-time utilization)		
Routine office visit	2	Assumption
Enema (additional colonoscopy and sigmoidoscopy if needed)*	1	Assume one-time treatment for GI toxicity
SD toxicity (per year)		
Pharmacologic treatment (sildenafil)	22	Cooke et al. (2005)

*GI, gastrointestinal; GU, genitourinary; IMRT, intensity-modulated radiation therapy; PSA, prostate specific antigen; PT, proton beam therapy; SBRT, stereotactic body radiation therapy; SD, sexual dysfunction*.

*Patients experiencing late GI toxicity were first treated with a 6-month course of enema in 70% of the cases. The remaining patients were assumed to undergo a colonoscopy followed by an average of three sigmoidoscopies and an additional 6-month course of enema. The cost is calculated as a one-time weighted average cost.

#### Costs

Table 5 displays the unit costs per unit of resource utilization that were used in the model. All unit costs in the table reflect Medicare payments. The SBRT treatment cost was based on nationally unadjusted Medicare rates using codes for robotic SBRT, and the remaining treatment costs were based on published sources. For the societal perspective, the age-specific cost per hour of time lost in treatment was based on 2011 estimates from the Bureau of Labor Statistics (2011). Additional patient costs, such as transportation costs, are not included in the societal perspective. From a lost work time perspective, we assumed that this wage value reflects the opportunity cost of time for both working and retired persons of the same age. The cost of routine monitoring and toxicity-related costs was applied annually for GU and SD whereas for GI a one-time cost was applied assuming that GI symptoms are less likely to be chronic. All costs were based on published sources and costs used in the analysis are expressed in 2011 US dollars, with costs and utilities discounted at 3.0% annually.

**Table 5:** **Model cost inputs**.

Cost parameters	Value	Source
**TREATMENT COST**		
SBRT	$20,889	Medicare rates – data on file, Accuray Inc.
IMRT	$28,805	Konski et al. (2006), MAG Mutual (2011)
PT	$65,250	Ollendorf et al. (2008)
**Work-time lost (cost per hour)**	$20	Bureau of Labor Statistics (2011)
**ROUTINE MONITORING**		
Urologist	$177	MAG Mutual (2011)
Office visit	$102	MAG Mutual (2011)
PSA	$103	MAG Mutual (2011)
**TOXICITY-RELATED**		
GU toxicity		
Daily pharmacologic treatment (oxybutynin)	$1	Red Book (2010)^‡^
Cystoscopy	$214	MAG Mutual (2011)
GI toxicity		
Enema (additional colonoscopy and sigmoidoscopy if needed)*	$259^†^	MAG Mutual (2011), Red Book (2010)^‡^
SD toxicity		
Daily pharmacologic treatment (sildenafil)	$12	Red Book (2010)^‡^

*GI, gastrointestinal; GU, genitourinary; IMRT, intensity-modulated radiation therapy; PSA, prostate specific antigen; PT, proton beam therapy; SBRT, stereotactic body radiation therapy; SD, sexual dysfunction*.

*Patients experiencing late GI toxicity were first treated with a 6-month course of enema in 70% of the cases. The remaining patients were assumed to undergo a colonoscopy followed by an average of three sigmoidoscopies and an additional 6-month course of enema. The cost is calculated as a one-time weighted average cost.

*^†^Assumes one-time treatment cost*.

*^‡^Costs are inflated to 2011 values based on the Consumer Price Index (Bureau of Labor Statistics, 2011)*.

### Analyses

#### Base-case model

Lifetime costs and QALYs per patient were calculated for each treatment. Lifetime costs were calculated by summing all costs associated with each health state that patients spend time in, and QALYs are calculated by summing the product of utilities associated with each health state and the time spent in the health state. The incremental cost-effectiveness ratio (ICER) value was calculated by first rank-ordering the treatment regimens by increasing cost and then comparing each strategy to the next less costly strategy by dividing the additional cost by additional benefit (QALY).

#### Sensitivity analyses

One-way sensitivity analysis was conducted to determine the parameters to which the ICER is most sensitive. One-way sensitivity analyses were performed by varying selected model parameters based on the 95% confidence interval (CI) of the base-case estimate, where available, while keeping all other parameters constant. CIs were not available for the costs used in the model, and so they were varied through a range of 75%–125% of the base-case estimate while keeping all other parameters constant. Probabilistic sensitivity analysis was also conducted to assess uncertainty in the cost-effectiveness analysis by determining the probability of a treatment being cost-effective at a threshold of $50,000/QALY. The probability estimates for toxicity and utility were assumed to follow a beta distribution [see Tables 2 and 3 for the standard errors (SE)], and cost parameters were assumed to follow a gamma distribution and the SE was calculated as 50% of the base-case default value.

An alternative cost scenario was tested by applying an annual cost for treating GI toxicity, instead of the one-time costs used in the base-case. In the absence of a SD toxicity rate for PT, an alternative toxicity scenario was tested by setting the SD toxicity for PT equal to SBRT. Finally, in threshold analyses, the toxicity of SBRT was varied from the base-case assumption to assess the impact of alternate toxicity rates on base-case results.

## Results

### Base-case model

Table 6 displays the results of the cost-effectiveness analyses from the payer and societal perspectives.

**Table 6:** **Cost-effectiveness results for all comparator treatments for the base-case**.

	Total per patient		Incremental		ICER Costs/QALY gained
	Costs	QALYs	Costs	QALYs	
**PAYER PERSPECTIVE**					
SBRT	$24,873	8.11	–	–	Reference
IMRT	$33,068	8.05	$8,195	−0.062	Dominated*
PT	$69,412	8.06	$44,539	−0.047	Dominated
**SOCIETAL PERSPECTIVE**^†^					
SBRT	$25,097	8.11	–	–	Reference
IMRT	$35,088	8.05	$9,991	−0.062	Dominated
PT	$71,657	8.06	$46,560	−0.047	Dominated

*ICER, incremental cost-effectiveness ratio; IMRT, intensity-modulated radiation therapy; QALY, quality-adjusted life year*.

*NOTE: Results are estimated for a patient 65 years old using Medicare reimbursement rates*.

*Dominated – higher cost and lower QALY.

*^†^Societal perspective also includes productivity costs owing to time spent in treatment*.

#### Payer perspective

SBRT was the least expensive option in terms of lifetime costs ($24,873), followed by IMRT ($33,068) and PT ($69,412). IMRT and PT were both more costly and yielded fewer QALYs when compared with SBRT (total QALYs: 8.11, 8.05, 8.06 for SBRT, IMRT, PT, respectively).

#### Societal perspective

When the value of lost time in treatment was included, SBRT remained the least expensive treatment option ($25,097), followed by IMRT ($35,088) and then PT ($71,657). Once again, IMRT and PT were both dominated by SBRT because they were more costly and yielded fewer QALYs when compared with SBRT (total QALYs: 8.11, 8.05, 8.06 for SBRT, IMRT, PT, respectively).

### Sensitivity analyses

**Figure 2:**
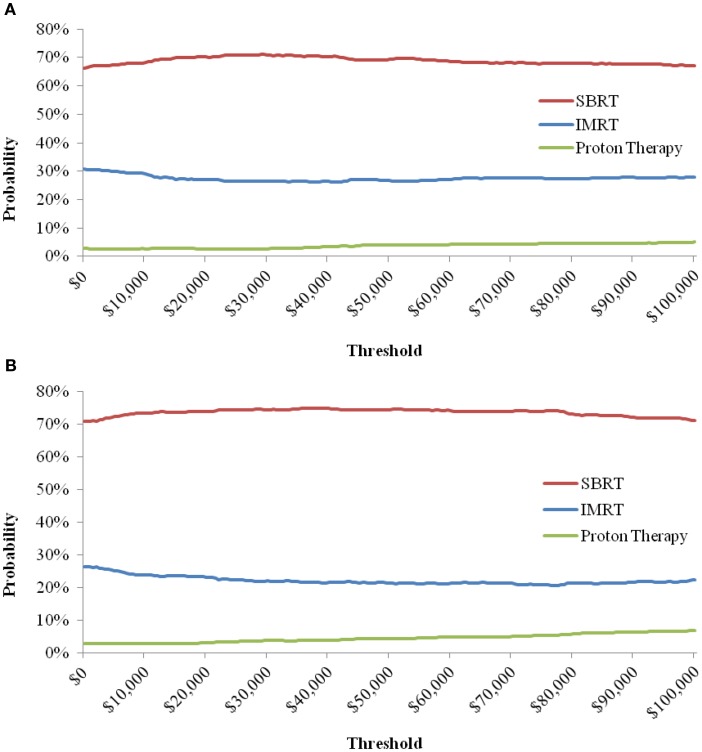
**Probability of SBRT being cost-effective when compared to IMRT and Proton Therapy, from the Payer (A) and Societal (B) Perspective**. Note: the cost-effectiveness acceptability curve shows the percentage of iterations in the probabilistic sensitivity analysis that are cost-effective at a certain threshold.

When the toxicity parameters were varied based on their CIs, and the costs by ±25%, the results did not change from the base-case in both payer and societal perspectives; SBRT was the dominant strategy, being less expensive with more QALYs compared to IMRT and PT. The probabilities of SBRT being cost-effective at $50,000/QALY from both the payer and societal perspective are presented in Table 7. The probabilities of SBRT being cost-effective compared to IMRT and PT are 75.1% and 94.1%, respectively, from the payer perspective; 75.1% and 95.5%, respectively, from the societal perspective. Figure 2 displays the probabilities of SBRT being cost-effective compared to IMRT and PT, from the payer (Figure 2A) and societal (Figure 2B) perspective.

**Table 7:** **Probability of SBRT being cost-effective at the $50,000/QALY threshold**.

SBRT Comparator	Payer perspective $50,000/QALY	Societal perspective $50,000/QALY
IMRT	75.1%	75.1%
PT	94.1%	95.5%

*IMRT, intensity-modulated radiation therapy; PT, proton beam therapy; QALY, quality-adjusted life year; SBRT, stereotactic body radiation therapy*.

The alternative cost scenario analysis using an annual cost for treating GI toxicity, instead of the one-time costs used in the base-case model, produced results that were similar to the base-case. Both IMRT and PT remained more costly and yielded fewer QALYs when compared with SBRT from either perspective. Also, Table 8 displays the results of an alternative toxicity scenario where SD toxicity for PT was set equal to SBRT, in contrast to the base-case model which set PT equal to IMRT. When the SD toxicity for PT was set equal to SBRT, then SBRT weakly dominates IMRT and no longer dominates PT.

**Table 8:** **Alternative sensitivity analysis with proton therapy toxicity set equal to SBRT toxicity**.

	Total per patient		Incremental		ICER Costs/QALY gained
	Costs	QALYs	Costs	QALYs	
**PAYER PERSPECTIVE**					
SBRT	$24,873	8.11	–	–	Reference
IMRT	$33,068	8.05	$8,195	−0.062	Weakly dominated*
PT	$69,094	8.17	$44,221	0.057	$13,755,207
**SOCIETAL PERSPECTIVE**^†^					
SBRT	$25,097	8.11	–	–	Reference
IMRT	$35,088	8.05	$9,991	−0.062	Weakly dominated
PT	$71,339	8.17	$46,242	0.057	$14,383,693

*ICER, incremental cost-effectiveness ratio; IMRT, intensity-modulated radiation therapy; PT, proton beam therapy; QALY, quality-adjusted life year; SBRT, stereotactic body radiation therapy*.

*NOTE: Results are estimated for a patient 65 years old using Medicare reimbursement rates*.

*Dominated – higher cost and lower QALY.

*^†^Societal perspective also includes productivity costs owing to time spent in treatment*.

In threshold analyses, when all three toxicity rates for SBRT were increased by 23% from the base-case, the total lifetime costs for SBRT was lower than for IMRT ($25,037 versus $33,068), and the QALYs for SBRT were slightly higher than for IMRT (8.051 versus 8.049). If all three toxicity rates for SBRT were increased by ≥24%, the QALYs for SBRT are lower than for IMRT. Furthermore, the threshold analyses showed that IMRT was only cost-effective compared to SBRT at a $100,000/QALY threshold when all three toxicity rates for SBRT were increased by 54% from the base-case rates, from a payer’s perspective. Similarly, from a societal perspective, IMRT was only found to be cost-effective at a $100,000/QALY threshold when the toxicity rates of SBRT were increased by at least 61% from the base-case rates.

Among the three toxicities included in the model, the SD toxicity rate for SBRT was found to be lower than for IMRT. If the SD toxicity rate for SBRT is increased by more than 43% from the base-case rate, then the QALY for SBRT was lower than for IMRT. From a payer’s perspective, the threshold analyses showed that IMRT was only cost-effective compared to SBRT at a $100,000/QALY threshold when the SD toxicity rate for SBRT was increased by 97% from the base-case rate. Similarly, from a societal perspective, IMRT was only cost-effective compared to SBRT at a $100,000/QALY threshold when the SD toxicity rate for SBRT was increased by 109% from the base-case rate.

## Discussion

In this study, we found that SBRT was less expensive than both IMRT and PT, with SBRT patients expecting to have a better quality of life owing to its more favorable toxicity profile. The probabilistic sensitivity analyses from both the payer and societal perspectives showed that, compared to the other treatments, SBRT is cost-effective at a $50,000/QALY threshold in 75%–96% of model simulations.

There is a continued trend of increasing utilization of more costly treatments for localized prostate cancer. From 2000 to 2008, the use of IMRT over the older 3D conformal radiation has increased from 28.7% to 81.7% (Nguyen et al., 2011). Since 2008, there has been an exponential increase in the number of proton facilities built in the United States – each of which costs approximately $200 million – with a corresponding increase in its use for prostate cancer treatment. Cost-effectiveness is one way to examine the “value” of different treatment options. Consistent with prior findings by Konski et al. (2007), we also demonstrate that proton therapy is unlikely to be cost-effective. Importantly, we find that newer technology does not always equate to costlier treatment. SBRT, because of an ability to shorten radiation treatment to five fractions, results in cost savings compared to IMRT and PT. If longer-term follow-up continues to demonstrate favorable toxicity and disease control outcomes for SBRT, then this study provides important data which may have policy implications.

This study has several limitations worth mentioning. There are limited data on SBRT for localized prostate cancer. In fact, since this study began, reports from five additional SBRT studies, each with varying numbers of patients and months of follow-up, have been published (Aluwini et al., 2010; Bolzicco et al., 2010; Kang et al., 2011; Townsend et al., 2011; Jabbari et al., 2012). None of these studies reached conclusions that differ substantially from the assumptions used in the present analysis (i.e., toxicity remains relatively low and disease control mirrors that obtained with other radiation therapy options). Further, the published literature on SBRT of localized prostate cancer has consisted mostly of patients treated with the CyberKnife Robotic Radiosurgery System (Accuray Incorporated, Sunnyvale, CA, USA; Friedland et al., 2009; King et al., 2009, 2012; Katz et al., 2010; Freeman and King, 2011). Two studies have been published which used conventional linear accelerators to deliver prostate SBRT (Madsen et al., 2007; Boike et al., 2011), but both used lower doses compared to current SBRT standards and therefore were not included in the model estimates. Inclusion of toxicity results from studies that used lower doses of SBRT may bias our results further in favor of SBRT. There is also a lack of data on the median time to resolution of long-term toxicity. Therefore, in the model, patients who develop long-term toxicity were assumed to remain in a health state reflective of their toxicity until they die.

In conclusion, based on the assumption that each treatment modality results in equivalent long-term disease control, results from this study suggest that SBRT is cost-effective, resulting in cost savings and improved quality-adjusted survival compared to IMRT and PT for the treatment of localized prostate cancer. Additional studies are needed to directly examine the comparative effectiveness of the different radiation treatments for localized prostate cancer. Given the lack of randomized trial data, this study provides important and novel information based on currently available published evidence, and contributes to an understanding of the comparative value of three types of external beam radiation treatments for localized prostate cancer.

## Authors’ contributions

Anju Parthan: Primary investigator, Narin Pruttivarasin: Primary economic modeler, Douglas C. A. Taylor: Study director, Vivek Pawar: Secondary economic modeler, Diane Davies: Health economist and author, Akash Bijlani: Health policy/economic specialist and author, Kristen Hassmiller Lich: Health policy specialist and author, Ronald Chen: Radiation oncologist and senior author.

## Conflict of Interest Statement

This study was funded by Accuray, Inc.
